# Corrosion Characteristics and Tensile Performance of Bolted Spherical Joints in Aggressive Environments

**DOI:** 10.3390/ma18102185

**Published:** 2025-05-09

**Authors:** Jianguo Li, Yanhong Li, Sheng Yang, Chenling Hao, Yun Yang, Chong Chen, Qingsong Zhou, Guanglin Yuan, Caifeng Lu

**Affiliations:** 1 Department of Civil Engineering, Shanxi Institute of Technology, Yangquan 045000, China; lijianguo@sxit.edu.cn; 2 Jiangsu Key Laboratory of Disaster Impact and Intelligent Prevention in Civil Engineering, School of Mechanics and Civil Engineering, China University of Mining & Technology, Xuzhou 221116, China; ts22030195p31@cumt.edu.cn (Y.L.); ts22030253p31@cumt.edu.cn (S.Y.); hcling@cumt.edu.cn (C.H.); ts24030245p31@cumt.edu.cn (Y.Y.); chenchong@cumt.edu.cn (C.C.); ygl65@cumt.edu.cn (G.Y.); 3 Building Materials Engineering Laboratory, Department of Architecture, Graduate School of Engineering, The University of Tokyo, Tokyo 113-8654, Japan; zhouqingsong@g.ecc.u-tokyo.ac.jp

**Keywords:** bolted spherical joint, corrosion, screwing depth, bearing capacity, failure mode

## Abstract

Bolted spherical joints (BSJs) are widely used in spatial grid structures owing to their clear force transmission paths and ease of on-site assembly. This study investigates the corrosion behavior and tensile performance of BSJs fabricated with #45 carbon steel joint spheres and 40Cr high-strength bolts (grade 10.9S) under chloride exposure under varying bolt screwing depths. Accelerated salt spray corrosion tests were conducted across different exposure cycles (20, 40, 60, and 80 cycles) and at screwing depths ranging from 0.8 d to 1.2 d, followed by uniaxial tensile testing. Results revealed that chloride-induced pitting corrosion was more pronounced on bolts than on joint spheres, with four distinct types of microscopic corrosion morphologies identified. Inadequate screwing depth (<1.0 d) led to pull-out failure, while greater depths (≥1.0 d) generally resulted in bolt fracture. Chloride exposure significantly reduced the ultimate tensile capacity of BSJs. For bolts with λ < 1.0, post-corrosion tensile strength dropped below the specification threshold, indicating a critical safety concern.

## 1. Introduction

Spatial grid structures are commonly used in large-span facilities such as industrial plants, sports arenas, and airport terminals [1,2,3]. Among the various joint types employed in these structures, bolted spherical joints (BSJs)—consisting of #45 carbon steel spheres and 40Cr alloy steel high-strength bolts—are especially favored due to their lightweight design, structural stiffness, and reliable force transmission.

**Figure 1:**
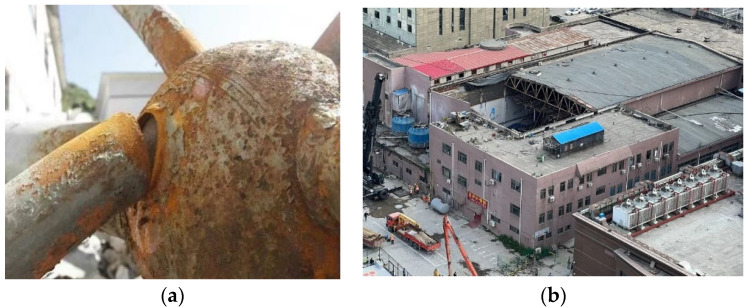
Engineering accident cases caused by corrosion. (**a**) Overall collapse of steel grid workshop. (**b**) Partial collapse of swimming pool roof.

However, BSJs are vulnerable to corrosion in environments with aggressive media, such as chlorine-rich atmospheres and marine aerosols [4,5,6,7]. In particular, chloride ions (Cl^−^) from coastal air or indoor swimming pool environments can penetrate protective coatings, initiating localized corrosion (e.g., pitting), and threatening the structural integrity of BSJs. According to the Chinese standard [8], the pool water in swimming pools needs to be regularly disinfected with liquid chlorine, which can cause corrosive chloride ions in the air inside the pool area and in the condensed water on the surface of a grid structure. Additionally, the corrosion of grid structures containing BSJs is generally severe in the marine atmospheric environment along the southeast coast of China, where the temperature and humidity are relatively high and corrosive chloride ions are present. Long-term coverage of a thin water film containing chloride ions on the grid structure causes damage to the protective layer on the surface of steel members and nodes, leading to corrosion. Once the bearing capacity of BSJ decreases due to chloride corrosion, the transmission path of grid structure will change, and the steel members or grid structure may lose their bearing capacity, leading to local structure damage or even continuous collapse of the structure. For example, in August 2013, a sudden overall collapse occurred in a steel truss workshop in Shanxi, China. The cause of the accident was attributed to severe corrosion of the truss members, nodes, and supports during long-term service, resulting in weakened cross-sections (Figure 1a). In April 2022, a swimming pool in Zhengzhou, China, experienced a partial collapse of its roof due to steel corrosion (Figure 1b). Therefore, attention should be paid to the BSJs in response to a corrosive environment, hence such disasters can be prevented.

At present, extensive research has been conducted on the mechanical properties [1,2,3], fatigue failure [9,10,11], and high-temperature performance degradation [12,13] of BSJs using experimental methods, theoretical analysis, and numerical simulations. For example, Liu [1] conducted a systematic study on the mechanical properties of large-diameter BSJs and their accessories in response to the increasing demand for such joints in China and developed the China Steel Structure Association Group Standard [14]. The research on fatigue performance mainly focuses on the ultra-low-cycle, low-cycle, medium-cycle, and high-cycle fatigue failure of bolts or BSJs with various sizes under constant-amplitude and variable-amplitude loading and has developed into the fatigue failure of grid structures connected by BSJs to steel pipe components [9,10,11].

Given the complexity of spatiotemporal variations in corrosion, a numerical simulation approach is more effective and efficient for studying corroded BSJs [4]. Tan et al. [5] studied the mechanical properties of corroded BSJs in a certain grid project under corrosive environments using full-scale experiments and further investigated the variation of corrosion degree with exposure time under atmospheric corrosive environments using numerical simulation methods. Here, in the finite element simulation, it is assumed that the bolts and joint sphere undergo uniform corrosion along the thickness direction simultaneously, without considering the actual possible local corrosion and pitting corrosion, which is obviously inconsistent with the actual situation. Yuan [4] conducted virtual uniaxial tensile tests on corroded BSJs with stochastic pitting corrosion on parametric finite element models, and discussed the load-displacement curves, axial stiffness, ultimate bearing capacity, and fracture morphology under different corrosion conditions. This study extends the simulation analysis of corroded BSJs by utilizing the research results of uncorroded BSJs, without physical test data of corroded BSJ to verify, which is similar to the shortcomings of reference [7].

The failure mode and bearing capacity of BSJs are closely related to the depth of high-strength bolts screwed into the joint sphere [2,15,16], and the design specifications also specify that the minimum screwing depth of high-strength bolts in BSJs should be at least 1.1 d [17], where d is the diameter of high-strength bolts. The fake tightened-up phenomenon of high-strength bolts is a common poor joint condition in BSJs due to deviations in prefabrication or on-site installation, which occurs quite often in practical engineering. Once the screwing depth of high-strength bolts is insufficient, the bearing capacity of BSJs decreases and brittle failure modes (such as bolt pull-out failure) may occur [2], which may lead to progressive collapse of spatial grid structures [18]. In addition, insufficient screwing depth of high-strength bolts can expose some bolt threads to the atmospheric environment, and condensed water containing corrosive medias on the surface of steel components can easily penetrate the connection gap between the bolts and the joint sphere, which is more likely to cause thread corrosion damage.

Even though corrosion damage accumulates continuously in BSJs serving in corrosive environments, and the fake tightened-up phenomenon of high-strength bolts is inevitable in grid steel structures, there was limited research on the tensile strength of BSJs with corrosion damage, and especially the influence of screwing depth on the bearing capacity of BSJs in corrosive environments remained unclear. To address these gaps, this study performed salt spray accelerated corrosion tests and uniaxial tensile tests on BSJs with varying screwing depths and corrosion cycles. The goal was to understand how corrosion morphology and severity affect mechanical performance, and to provide design recommendations for corrosion-prone environments. The corrosion morphology, failure mode, load-displacement curve, stiffness, and ultimate bearing capacity of BSJs with different screwing depths under different corrosion conditions were obtained and discussed.

## 2. Experimental Overview

### 2.1. Design of BSJ

**Figure 2:**
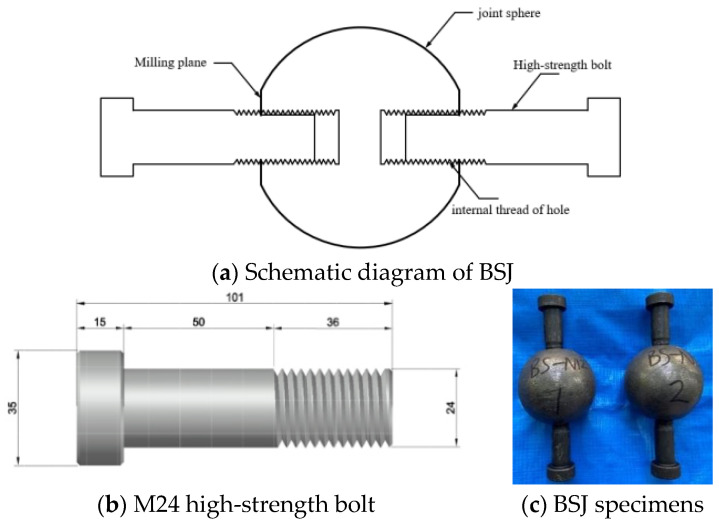
Design of BSJs.

In order to investigate the tensile performance of BSJs with different screwing depths in a chloride exposure environment, BSJs with double holes were designed simplistically according to the basic requirements of relevant technical regulations [17,19,20] to ensure the tension transmission path, as shown in Figure 2. The joint sphere and high-strength bolts adopt the commonly used dimensions and materials in spatial grid structures, where the joint sphere with a diameter of 100 mm was made of #45 carbon steel, and M24 high-strength bolts (grade 10.9S) were made of #40Cr alloy steel with thread pitch of 3 mm. The material properties of joint spheres and high-strength bolts are listed in Table 1a,b.

The production of BSJs takes into account different screwing depths (*K* = *λd*) of high-strength bolts, where d is the nominal diameter of high-strength bolts, known as d = 24 mm; and λ is the depth coefficient of high-strength bolts screwed into the joint sphere, which is taken as 0.8, 0.9, 1.0, 1.1, and 1.2 in this study. The screwing depth was measured using a digital vernier caliper and marked with a white mark before assembling BSJs, then the bolts were screwed into joint sphere using a wrench to the corresponding mark.

### 2.2. Chloride Exposure

Two major test methods, i.e., exposure to natural corrosive environments and accelerated corrosion in a laboratory, are adopted in experimental studies. Although exposure experiments in natural corrosive environments can accurately reflect the corrosion behavior of steel in marine atmospheric environments, this method has many disadvantages such as long test cycles, uncontrollable corrosion factors, and almost impossible reproducibility of test results. Therefore, the laboratory accelerated test is accepted to simulate the actual service corrosion and to study the corrosion mechanism and mechanical properties of corroded BSJs [4,5].

**Figure 3:**
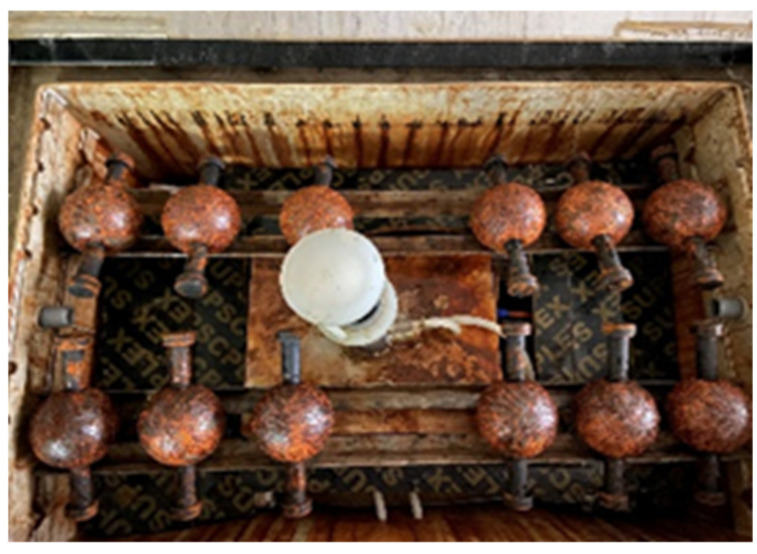
Salt spraying accelerated corrosion test.

An accelerated corrosion test that includes a periodic salt spraying–drying process was designed to simulate the corrosion behavior of BSJs in marine atmospheric corrosion environments. The procedure mainly contained several steps: (1) Set the salt spraying time to 9 h; (2) dry at a constant temperature for 9 h; (3) alternate between dry and wet cycles; (4) conduct a corrosion test on BSJs for a predetermined number of cycles. In this study, the corrosion cycle times were set to 20, 40, 60, and 80, respectively, and Figure 3 shows a photo of the salt spray test site. The experimental environment parameters are automatically controlled by the ASR-90A salt spray testing machine, as shown in Table 2.

Prior to the corrosion tests, the surface oxide layer of each BSJ specimen was removed using an angle grinder. The specimens were then degreased with acetone, air-dried, and their initial mass (*m*_0_) was recorded using a precision electronic balance. Upon completion of the designated corrosion cycles, the BSJ specimens were removed, and their surface corrosion morphologies were visually examined. Subsequently, corrosion products were chemically removed in accordance with the GBT16545-2015 standard [23], and the post-corrosion mass (*m*_1_) was measured. Then, we weighed the remaining mass m_1_ after corrosion again. The corrosion quality loss rate *ω* of BSJs can be calculated using Formula (1):

(1) $$\omega =\frac{m_{0}-m_{1}}{m_{0}}\times 100\%$$

### 2.3. Experimental Grouping

The test groups are shown in Table 3. This experiment was conducted in two batches. The first batch focused on studying the influence of corrosive environment on the mechanical properties of BSJs, where the screwing depth of bolts is uniformly set to 1.1 d. The second batch investigated the influence of the screwing depths on the mechanical properties of BSJs in corrosive environments, where the number of salt spraying corrosion cycles is uniformly set to 60. The 60-cycle corrosion condition in Batch II was selected based on observations from Batch I, where it showed clearly distinguishable corrosion effects while maintaining mechanical integrity for comparative analysis.

### 2.4. Uniaxial Tensile Test

**Figure 4:**
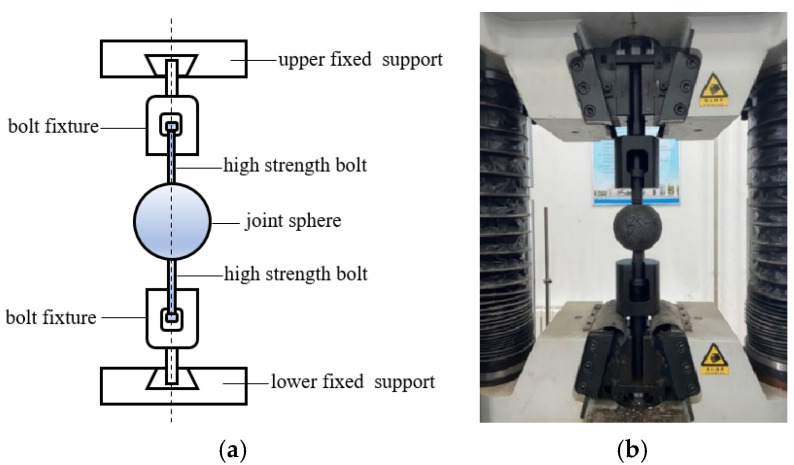
Uniaxial loading test. (**a**) Schematic diagram of loading device. (**b**) 60t hydraulic tensile testing machine.

Both uncorroded and corroded BSJs with different screwing depths were subjected to uniaxial tensile tests using a 60t hydraulic tensile testing machine. The high-strength bolts were clamped at both ends using self-made high-strength fixtures, as shown in Figure 4. The tensile testing was conducted following the GB/T228-2010 standard [24], with the loading process divided into pre-loading and formal loading phases. A displacement-controlled loading rate of 2.5 mm/min was applied. Throughout the test, axial force and end displacement were continuously recorded by the testing system until specimen failure.

## 3. Results and Discussion

### 3.1. Corrosion Results and Discussion

**Figure 5:**
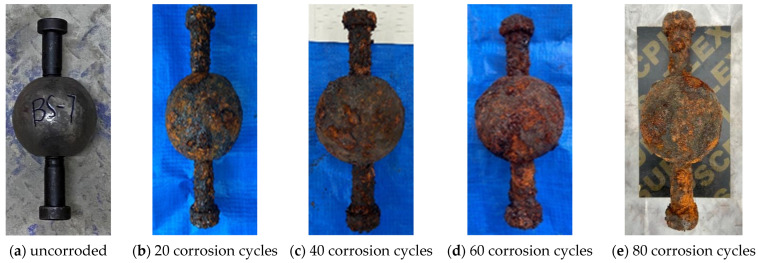
BSJ specimens reaching the predetermined corrosion cycles.

**Figure 6:**
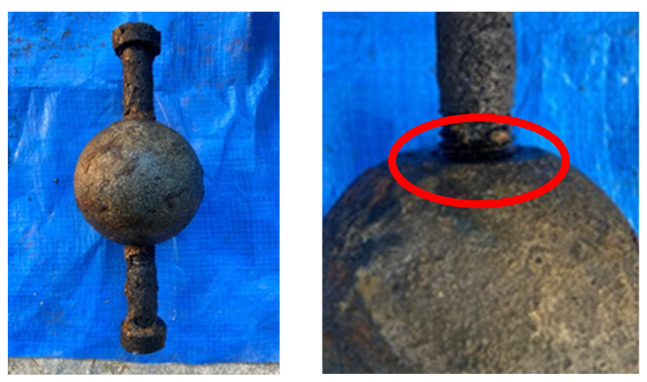
Specimen BS-3 after removing corrosion products.

In the chloride corrosion environment, the surface of BSJs became gradually enveloped by corrosion products, and the junction between joint sphere and high-strength bolts was gradually sealed by accumulated corrosion products. The color of corrosion products changed from initially light yellow to brown, and finally to reddish brown, as shown in Figure 5, due to the gradual change in the composition of the rust layer [25]. After using the acid washing method to remove corrosion products from the corroded BSJs, it was found that there were obvious corrosion pits on the surfaces of joint sphere and high-strength bolt. Figure 6 shows the BSJ that had undergone 60 corrosion cycles (BS-03). The small corrosion pits on the surface of joint sphere were densely distributed, while the corrosion pits on the surface of the high-strength bolt were deeper and distributed in patches, indicating that the corrosion of the high-strength bolt was relatively more severe. In addition, the surface of high-strength bolts at exposed threads is uneven, and the effective cross-sectional size was significantly reduced, and the originally smooth and full thread profile no longer existed.

**Figure 7:**
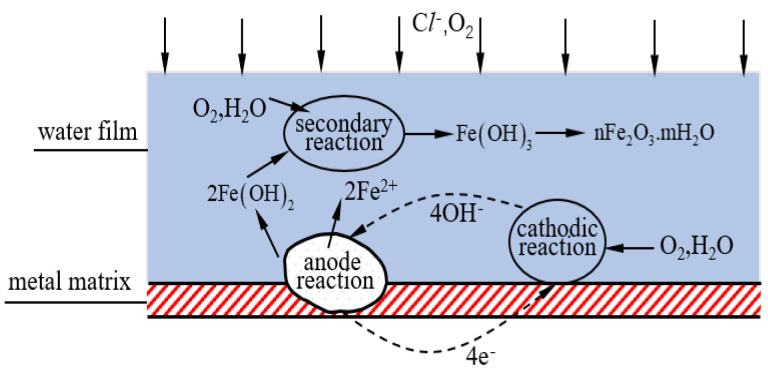
Schematic diagram of pitting corrosion mechanism.

In the salt spraying corrosion test, water film containing chloride ions and dissolved oxygen attached to the surface of BSJs serves as an electrolyte, and the formation of many micro corrosion cells causes pitting corrosion of BSJs [26,27]. Pitting corrosion usually includes two stages: pitting occurrence and pitting growth. There are currently two theories for pitting corrosion occurrence, namely the passivation film damage theory and the adsorption theory. Once the corrosion holes on the surface of BSJs are formed, the active metal matrix inside the holes acts as the anode for an oxidation reaction (Equation (2)), while the dissolved oxygen in the water film undergoes reduction reaction as the cathode (Equation (3)). As a result, many micro electrochemical corrosion cells are formed inside and outside the corrosion holes, as shown in Figure 7. The pH value outside the corroded pore increases, and the metal ions undergo secondary reactions, as shown in Equations (4) and (5). As corrosion progresses, Cl^−^outside the pores continuously migrates into the pores under the action of the corrosion microbattery, promoting self-catalytic corrosion reactions inside the pores and continuously consuming the metal substrate to generate more rust products, resulting in the appearance of many corrosion pits on the surface of the cleaned BSJs.

(2) $$\mathrm{Fe}\to Fe^{2+}+2e^{-}$$

(3) $$O_{2}+2H_{2}O+4e^{-}\to 4OH^{-}$$

(4) $$Fe^{2+}+2OH^{-}\to Fe{\left(OH\right)}_{2}$$

(5) $$4Fe{\left(OH\right)}_{2}+O_{2}+2H_{2}O\to 4Fe{\left(OH\right)}_{3}$$

**Figure 8:**
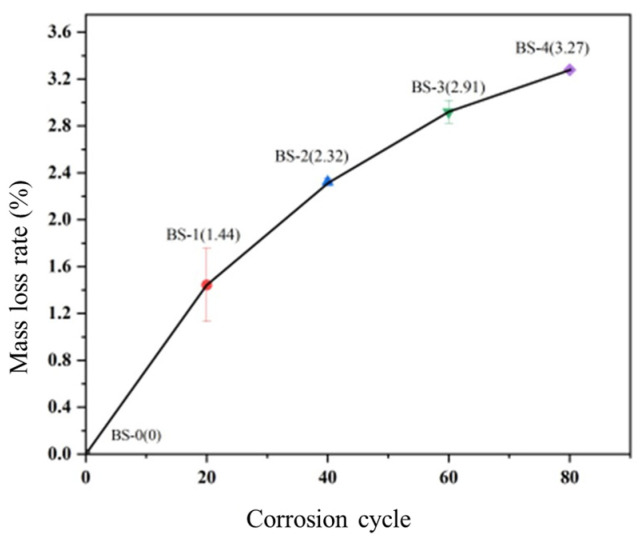
Mass loss rate of corroded BSJs under different corrosion cycles.

The quality loss rate of corroded BSJs, as shown in Figure 8, gradually increases with the extension of corrosion cycles, but the increase rate gradually slows down. Although the continuous accumulation of rust products on the surface of BSJs delays the process of direct corrosion of the metal substrate by Cl^−^, the corrosion progression will persist in occurring deep into the corrosion pits due to the strong penetration of Cl^−^, resulting in increased quality loss.

**Figure 9:**
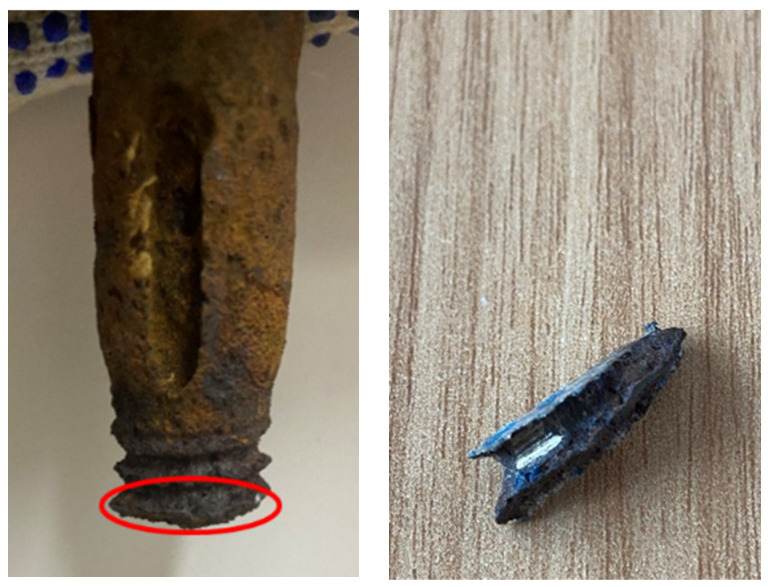
Sample taken from the exposed thread of fractured bolt.

**Figure 10:**
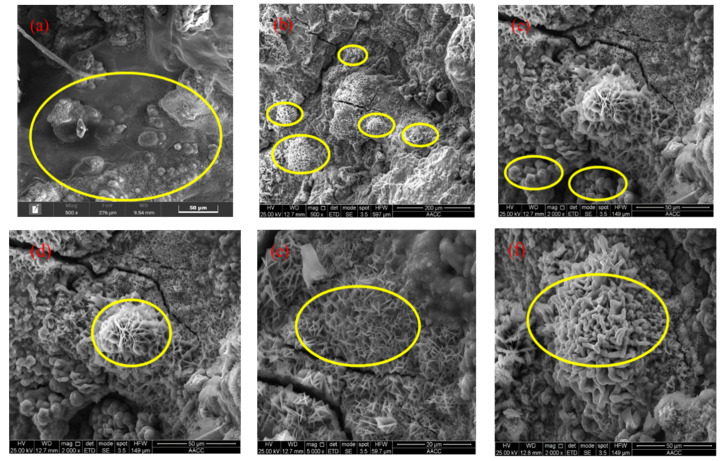
Microscopic corrosion morphologies.

A small section of the exposed threads was cut from the bolt after fracture failure in the axial tensile test described later (Figure 9), and the microstructure was observed using a Quanta 250 environmental scanning electron microscope. There are many cracks and protruding rust products on the surface of the corroded sample (Figure 10a), and there are also many “nest” shaped structural morphologies on the rust products (Figure 10b). Four typical micro corrosion morphologies can be clearly observed in the corrosion area, namely cotton-shaped (Figure 10c), flower-shaped (Figure 10d), needle-shaped (Figure 10e), and rod-shaped (Figure 10f).

**Figure 11:**
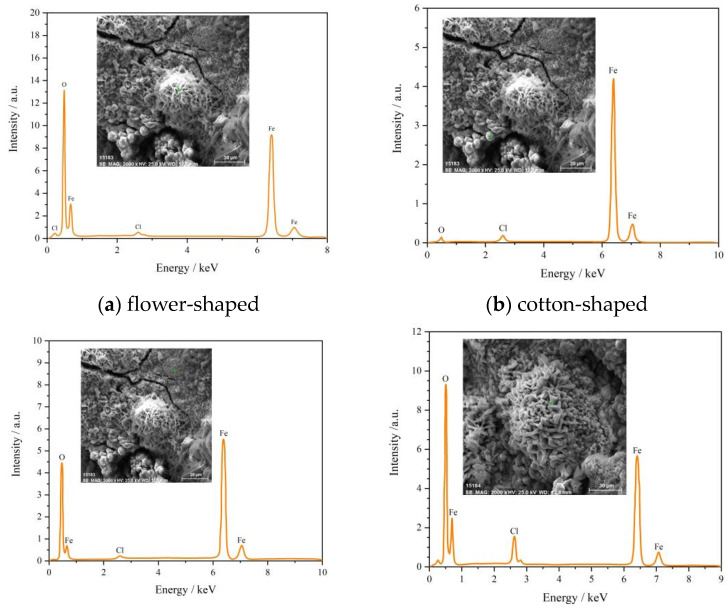
Energy spectrum analysis results of four typical micro corrosion morphologies.

**Figure 12:**
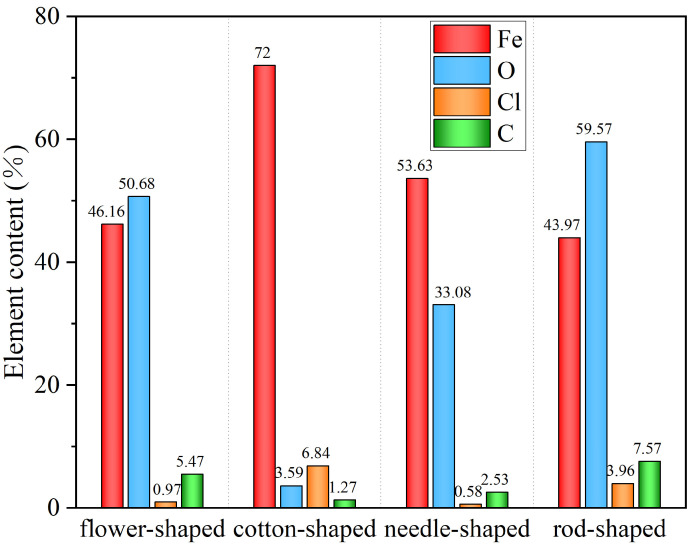
Relative element content (elemental content is derived from surface EDS measurements and may not sum to 100% due to surface oxides, light-element detection limits, and sample preparation effects. The results are intended for qualitative comparison only).

We selected one point from each of the four corrosion morphology regions for energy spectrum analysis (Figure 11) and listed the analysis results of element relative content in Figure 12. The flower-shaped corrosion morphology had loose, sheet-like rust products on the petals, high content of element O, low content of element Cl, and was mainly composed of Fe_2_O_3_ [28]. The cotton-shaped corrosion morphology can be regarded as the exposed flower style after the loose rust product falls off from the flower-shaped corrosion morphology, with low content of element O, high content of element Fe, and it is mainly composed of FeCl_3_. When extending longitudinally from the style to the petals, the content of element Cl gradually decreased while the content of element O increased significantly, which was consistent with the research conclusion of Wan et al. [29]. Previous studies have shown that the main components of corrosion products in needle-shaped and rod-shaped corrosion morphologies are γ-FeOOH and β-FeOOH, respectively [27]. The content of Fe element in the rod-shaped corrosion morphology decreased compared to the needle-shaped morphology, while the content of element O and Cl increased, indicating that the increase in Cl content was beneficial for the generation of β-FeOOH [30]. In summary, chloride-induced pitting corrosion plays a dominant role in the degradation of BSJs. The corrosion severity was higher in exposed bolt threads, directly influencing tensile strength reduction discussed in the following sections.

### 3.2. Failure Modes

**Figure 13:**
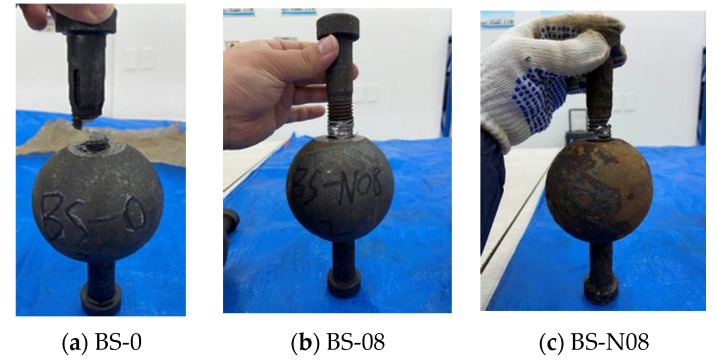
Failure mode of BSJs.

The failure modes of uniaxial tensile tests on all uncorroded and corroded BSJs with different screwing depths are shown in Figure 13 and Table 4. Two different failure modes of the tested BSJs subjected to axial tension were observed, namely bolt fracture failure and bolt pull-out failure. ① Bolt fracture failure: When the high-strength bolt is screwed deep enough into the joint sphere, it reaches its ultimate strength under tension and fractures, which is the main failure mode of tension BSJs. ② Bolt pull-out failure: When the depth of high-strength bolts screwed into the joint sphere is relatively small, the occlusion damage between the high-strength bolts and the joint sphere causes a decrease in the shear bearing capacity of the threads inside the joint sphere, resulting in the high-strength bolts being pulled out of the joint sphere, thus also known as thread failure. Bolt pull-out failure occurs without obvious signs, which belongs to brittle failure.

Carefully observing the failure characteristics of all BSJs in Figure 13, it can be seen that the following are true: ① For the uncorroded and corroded BSJs with screwing depth 1.1 d (BS-0~BS-4), the failure type is always bolt fracture failure accompanied by a loud fracture sound. It was quite consistent for all tested BSJs that the fracture location started at the root of the exposed threads of high-strength bolts where stress concentration existed [31], with a sharp fracture section at a certain angle to the axial direction of bolts. Although the corrosive environment did not change the failure mode of BSJs, the fracture surfaces exhibited increasing roughness with the extension of the corrosion cycles. The closer to the fracture location, the more obvious the crack at the bottom of the threads is, but the fractured bolts did not show significant necking. ② For the uncorroded BSJs with the screwing depth coefficient λ < 1.0 (BS-08, BS-09), the failure mode was bolt pull-out failure, which was quite consistent with the previous literature [2]. When the depth coefficient was λ ≥ 1.0 (BS-10, BS-11, BS-12), the failure mode of BSJs was bolt fracture failure, and the initial fracture position was compltely located at the root of the exposed threads as described earlier, and the fractured bolt had no significant necking phenomenon. Except for the fracture section of specimen BS-12, which was approximately perpendicular to the bolt axis, the other fracture sections were approximately at a 45° angle to the bolt axis. This once again stated that in order to obtain the design strength of BSJs, the screwing depth of high-strength bolts should not be less than the bolt diameter, as specified in Chinese standards with the screwing depth of 1.1 d [17,19]. ③ For corroded BSJs with different screwing depths of high-strength bolts after 60 corrosion cycles, as shown in BS-N08~BS-N12, bolt fracture failure occurred, and the fracture section was more uneven than that of the uncorroded BSJs. Here, when the depth coefficient of the high-strength bolts is λ < 1.0 (BS-N08, BS-N09), the corroded BSJs did not experience bolt pull-out failure like the uncorroded BSJs. This may be due to severe degradation of the exposed threads of the bolt by chloride ions, reducing the effective cross-sectional area of the bolt at the exposed threads. As a result, the tensile bearing capacity of high-strength bolts decreased, and the high-strength bolts first fractured before the threads in joint sphere reach the ultimate shear bearing capacity.

In addition to classifying failure modes, Figure 13 and Table 4 also reflect localized corrosion features that contributed to mechanical failure. For instance, fractured bolts in corroded BSJs (e.g., BS-N08 to BS-N12) exhibit rough and uneven surfaces at the thread root, contrasting with the smoother fractures in uncorroded samples. These morphological features visually suggest that chloride-induced pitting and corrosion-assisted cracking played a key role in initiating failure. The correlation between corrosion severity and fracture characteristics further supports the interpretation of brittle failure patterns discussed above.

### 3.3. Force–Displacement Curves and Tensile Bearing Capacity

**Figure 14:**
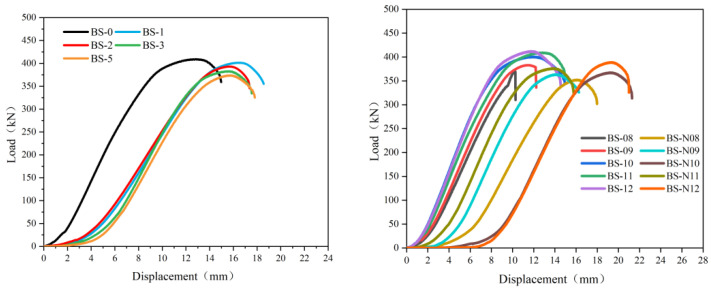
Load–displacement curves of BSJs.

Figure 14 shows the load–displacement curves obtained in the tension tests for all uncorroded and corroded BSJs with different screwing depths, which exhibit distinct nonlinear characteristics closely related not only to chloride corrosion but also to the screwing depth of high-strength bolts. ① For uncorroded BSJs with different screwing depths, a high degree of consistency was maintained between displacement and load during the initial loading period. Compared to the uncorroded specimen, the corroded BSJs experienced a much slower increase in load during the initial stage of axial tensile testing (displacement less than 2 mm) due to residual loose rust products at the bottom of nuts, resulting in relatively larger displacement corresponding to the peak point of curves. ② For uncorroded BSJs with the screwing depth of high-strength bolts K<1.0d (BS-08 and BS-09)), the load–displacement curves dropped vertically after the applied tension reached the peak points, and the corresponding failure mode was bolt pull-out failure (Figure 13b,c). However, the load–displacement curves for other BSJs decreased gradually after the applied tension reached the peak points (bolt fracture failure), and the peak points of curves (ultimate bearing capacity) gradually decreased with the extension of corrosion cycles.

**Figure 15:**
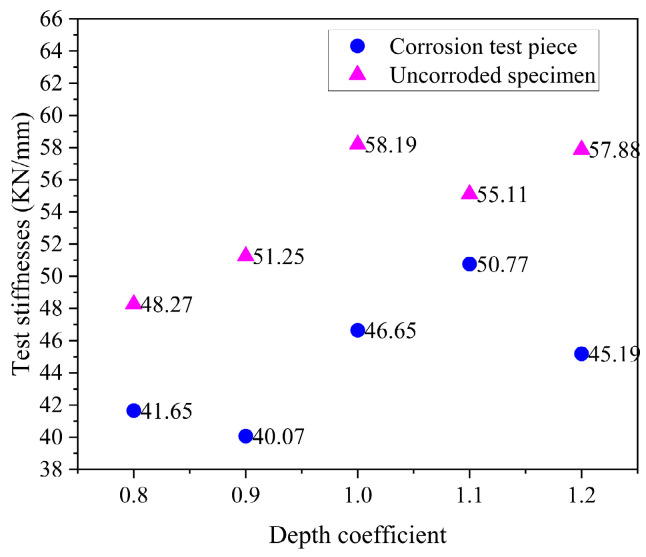
Calculated stiffnesses of tested BSJs.

The tested stiffnesses of corroded and uncorroded BSJs with different screwing depths are shown in Figure 15, which were calculated as the slopes of load–displacement curves between 30% and 50% of displacement at the peak points. Chloride corrosion reduced the calculated stiffness of BSJs. For uncorroded and corroded BSJs, the stiffness was significantly lower than that of the control group (depth coefficient 1.1) when the depth coefficient was λ < 1.0, due to the relative slip between the threads of high-strength bolts and the joint sphere.

**Figure 16:**
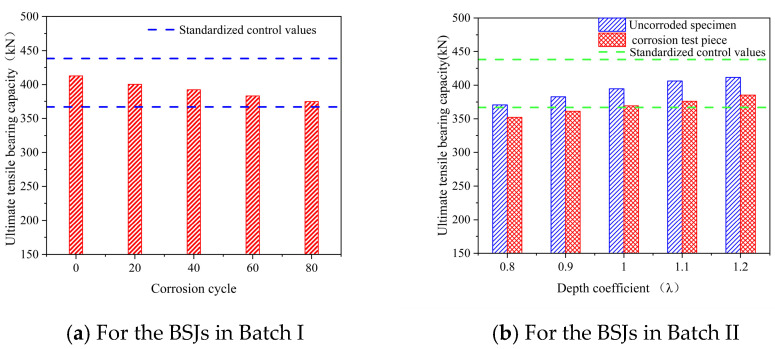
Ultimate tensile bearing capacity.

Figure 16 shows the tensile bearing capacity obtained in the tension tests for all uncorroded and corroded BSJs with different screwing depths, where the two dashed lines represent the control values in relevant specifications [17]. Obviously, the ultimate tensile bearing capacity of the BSJs with the screwing depth 1.1 d was within the range of the control values and gradually decreased with the extension of corrosion cycles (Figure 16a). After 20, 40, 60, and 80 corrosion cycles, the ultimate tensile bearing capacity of BSJs decreased by 2.96%, 4.90%, 7.10%, and 9.21%, respectively. Additionally, reducing the screwing depths of high-strength bolts lowered the ultimate tensile bearing capacity of BSJs (Figure 16b). It was particularly important to note that the ultimate tensile bearing capacity of BSJs with a depth coefficient λ < 1.0 after 60 corrosion cycles by chloride ions failed to meet the minimum control value requirements. In the case of bolt fracture failure, the bearing capacity of BSJs was not only related to the strength of high-strength bolts, but also closely related to the effective cross-sectional area of exposed threads. Corrosion environment would reduce the effective cross-sectional area of exposed threads, resulting in a decrease in bearing capacity of the BSJs.

These results indicate a direct correlation between corrosion severity and tensile performance degradation. Chloride-induced pitting corrosion not only reduces the effective cross-sectional area of exposed threads but also promotes microcracks at stress-concentration zones, thereby lowering the tensile strength and altering the failure mode from ductile pull-out to brittle fracture. This explains why corroded specimens with insufficient screwing depth (λ < 1.0) exhibited fracture failure rather than pull-out, which was observed in uncorroded counterparts. The coupling effect of corrosion and inadequate bolt engagement significantly undermines the safety margin of BSJs.

## 4. Conclusions

This study systematically investigated the effects of chloride corrosion and screwing depth on the corrosion morphology, tensile failure modes, and bearing capacity of bolted spherical joints (BSJs) through salt spray accelerated corrosion and uniaxial tensile tests. The main findings are as follows:

(1) In chloride-rich environments, BSJs experience progressive electrochemical corrosion, with pitting corrosion more severe on high-strength bolts than on joint spheres. Corrosion product color and mass loss increased with longer exposure.
(2) Four distinct microscopic corrosion morphologies—flower-shaped, cotton-shaped, needle-shaped, and rod-shaped—were identified. Their composition was influenced by variations in elemental oxygen and chlorine content.
(3) For BSJs with a screwing depth of 1.1 d, the failure mode remained bolt fracture, regardless of corrosion. Fractures consistently initiated at the root of exposed threads, where stress concentration occurred. Corroded samples exhibited rougher fracture surfaces.
(4) Uncorroded BSJs with λ < 1.0 showed pull-out failure, whereas corroded counterparts underwent bolt fracture. This shift was attributed to corrosion-induced reduction in the effective cross-sectional area of exposed threads.
(5) The ultimate tensile capacity of BSJs decreased with both increasing corrosion exposure and decreasing screwing depth. BSJs with λ < 1.0 after 60 corrosion cycles failed to meet the minimum bearing capacity specified by design standards.
(6) Although this study focused on as-received BSJ specimens without additional treatments, future research should explore how thermal (e.g., tempering) and chemical (e.g., passivation and coating) treatments influence the corrosion resistance and tensile performance. Such treatments could potentially mitigate pitting corrosion and delay failure under chloride environments.

## Figures and Tables

**Table 1:** (**a**): Properties of high-strength bolt and joint sphere. (**b**): Chemical composition (wt.%) of #45 carbon steel and 40Cr alloy steel according to GB/T 699-2015 [21] and GB/T 3077-2015 [22].

(**a**)								
**Category**	**Elastic Modulus** **(MPa)**	**Yield Strength** **(MPa)**	**Ultimate Strength** **(MPa)**		**Ultimate Strain**		**Poisson’s Ratio**	
High-strength bolt	2.1 × 10^5^	940	1040		0.10		0.3	
Joint sphere	2.1 × 10^5^	355	600		0.16		0.3	
(**b**)								
**Material**	**C**	**Si**	**Mn**	**Cr**	**Ni**	**Mo**	**S**	**P**
#45 Carbon Steel	0.42–0.50	0.17–0.37	0.50–0.80	≤0.25	≤0.30	—	≤0.035	≤0.035
40Cr Alloy Steel	0.37–0.44	0.17–0.37	0.50–0.80	0.80–1.10	≤0.30	≤0.10	≤0.035	≤0.035

**Table 2:** Parameters of accelerated corrosion test with periodic salt spray.

Temperature inside the test chamber	35 °C ± 1 °C
Relative humidity during spray	≥95%
NaCl solution concentration (mass fraction)	5%
Solution pH value	6.5–7.2
Salt spray deposition amount	1–2 mL/(80 cm^2^·h)
Pressure control during spray	0.2 MPa
Cycle design of periodic spray	Salt spraying of 9 h, and drying for 9 h

**Table 3:** Experimental grouping.

Test in Batches	Sample Number	Depth Coefficient (λ)	Numbers of Corrosion Cycles
Batch I	BS-0 (control group)	1.1	—
	BS-1	1.1	20
	BS-2	1.1	40
	BS-3	1.1	60
	BS-4	1.1	80
Batch II	BS-08	0.8	—
	BS-09	0.9	—
	BS-10	1.0	—
	BS-11	1.1	—
	BS-12	1.2	—
	BS-N08	0.8	60
	BS-N09	0.9	60
	BS-N10	1.0	60
	BS-N11	1.1	60
	BS-N12	1.2	60

**Table 4:** Summary of failure modes of BSJ specimens under different corrosion cycles and screwing depths.

Specimen ID	Corrosion Cycles	Screwing Depth λ	Failure Mode
BS-0	0	1.1	Bolt fracture
BS-1	20	1.1	Bolt fracture
BS-2	40	1.1	Bolt fracture
BS-3	60	1.1	Bolt fracture
BS-4	80	1.1	Bolt fracture
BS-08	0	0.8	Bolt pull-out
BS-09	0	0.9	Bolt pull-out
BS-10	0	1.0	Bolt fracture
BS-11	0	1.1	Bolt fracture
BS-12	0	1.2	Bolt fracture
BS-N08	60	0.8	Bolt fracture (corroded)
BS-N09	60	0.9	Bolt fracture (corroded)
BS-N10	60	1.0	Bolt fracture (corroded)
BS-N11	60	1.1	Bolt fracture (corroded)
BS-N12	60	1.2	Bolt fracture (corroded)

## Data Availability

The original contributions presented in the study are included in the article, further inquiries can be directed to the corresponding author.
